# Determinants of Pregnancy-Related Anxiety among Women Attending Antenatal Checkup at Public Health Institutions in Debre Markos Town, Ethiopia

**DOI:** 10.1155/2022/6935609

**Published:** 2022-08-08

**Authors:** Marta Yimam Abegaz, Haymanot Alem Muche, Getie Lake Aynalem

**Affiliations:** Department of Clinical Midwifery, School of Midwifery, College of Medicine and Health Sciences, University of Gondar, Gondar, Ethiopia

## Abstract

**Background:**

Pregnancy-related anxiety has been associated with many pregnancy adverse outcomes including preterm birth, low birth weight, postpartum depression, and resulting in long-term sequels on the child's emotional, cognitive, and behavioral development. This study is aimed at assessing the magnitude of pregnancy-related anxiety and associated factors among pregnant women attending antenatal checkup at Debre Markos town public health institutions, Northwest Ethiopia.

**Methods:**

An institution-based cross-sectional study was conducted among 423 pregnant women at Debre Markos town, Northwest Ethiopia, from February 1^st^ to March 30^th^, 2021. A systematic random sampling technique was used to select the study participants. Data were collected sing a structured, pretested, and interviewer-administered questionnaire. The collected data were entered with Epi-data version 4.6 and then exported to SPSS version 23. Both bivariable and multivariable logistic regression analyses were undertaken to identify significantly associated variables with pregnancy-related anxiety. The adjusted odds ratio (AOR) with its 95% confidence interval (CI) at a *p* value of ≤0.05 was used to claim statistical association.

**Result:**

In this study, a total of 408 pregnant women participated, giving a 96.4% response rate. The prevalence of pregnancy-related anxiety was found to be 43.9% (95% CI: 39.5, 49.2). Having no formal education (AOR = 3.37; 95% CI: 1.32, 8.58), primigravida (AOR = 1.94; 95% CI: 1.17, 3.24), intimate partner violence (AOR = 2.88; 95% CI: 1.47, 5.64), and poor social support (AOR = 2.05; 95% CI: 1.18, 3.56) was significantly associated with pregnancy-related anxiety.

**Conclusion:**

In this study, the prevalence of pregnancy-related anxiety was found to be high when compared to other study findings. The regional educational department should give emphasis for gender pedagogies which pay attention to the specific learning needs of girls. In addition, interventions on violence against women and social support for the women may reduce the problem.

## 1. Introduction

Pregnancy is a time of joy and mental wellbeing, but susceptibility to different mental disorders like anxiety and depression is also quite common for many women [1]. Pregnancy-related anxiety (PRA) is a fear, worry, and disrupting sense of peace related to the health of the woman, the baby, the pregnancy, and the delivery [2, 3]. It is a psychological stressor during pregnancy relatively distinctive from depression and general anxiety [4, 5].

It is among the common maternal mental health problems during pregnancy [6–8]. Globally, 11.4% [7] to 63% [1] of women experience anxiety during pregnancy. In different parts of Africa, its prevalence has also been reported as 26% in Nigeria [9], 44.9% in Benin [10], 15.2% in South Africa [11], and 25% in Tanzania [12]. It is an important public health concern that contains both personal characteristics or traits and environmentally influenced states [13, 14].

Pregnancy-related anxiety negatively affects maternal, fetal, neonatal, and child health during the antenatal, postnatal, and childhood period [5, 14–17]. It increases the risk of maternal antenatal depression, preeclampsia/eclampsia, prolonged labor, and unplanned cesarean section rate [16, 18]. Besides, it is a risk factor for various fetal developmental problems like oligohydramnios, intrauterine growth restriction, diminished placental perfusion, adverse fetal neurodevelopment, low birth weight, and preterm birth [14, 17, 19–21]. Additionally, PRA increases the risk of postpartum depression [5, 15] and perceived disability regarding everyday activity limitations and participation restrictions [7]. Moreover, it is a predictor of poor maternal-fetal bonding and poor maternal nursing care including decreased likelihood of breastfeeding and lower compliance with immunization schedules which in turn results in child growth restriction, severe malnutrition, and diarrhea [15]. PRA has also a long-term impact on a child's emotional, cognitive, and behavioral development ^14.^

Many predictive factors of PRA such as the educational status of the women, pregnancy complications, social support, intimate partner violence, and partner factors can be identified during routine prenatal care. So, having a good understanding of the prevalence and factors associated with PRA aids in elaborating preventive antenatal care to prevent it by simple and modified ways like awareness creation, partner counseling, and support [8, 22].

Even though PRA is a common mental disorder and has an overall negative impact on maternal wellbeing, their children, and their families, awareness about it is low since its symptoms overlap with the pregnancy. So, it remains without treatment [15, 23]. It is often a neglected problem and has been given lower priority particularly in lower and middle-income countries including Ethiopia [24]. Since preventive medicine is best, knowing about the prevalence and associated factors of pregnancy related anxiety is important in order to prevent the short and long term consequences of PRA on the maternal and child health. But as to the researchers best search, there is no study conducted on pregnancy related anxiety at the study area as well as Ethiopia at large.

Therefore, this institution-based cross-sectional study was aimed at determining the prevalence of pregnancy-related anxiety and associated factors among women attending antenatal care in Debre Markos town public health institutions, Northwest Ethiopia.

## 2. Methods

### 2.1. Study Design, Period, and Setting

An institution-based cross-sectional study was conducted from February 1^st^ to March 30^th^; 2021. This study was conducted in Debre Markos town public health institutions. The town is located in the East Gojjam zone, Amhara regional state, Northwest Ethiopia. It is 299 km far from Addis Ababa (the capital city of Ethiopia) and 265 km from Bahir Dar (the capital city of Amhara Regional state). According to the Population projection of Ethiopia for all regions at Woreda level from 2014 to 2017, the total population of the town is estimated to be 92,470, among these 46,738 are females [25]. Debre Markos town has one comprehensive specialized hospital and three public health centers. All the four public health institutions in the town are providing antenatal care (ANC) services. From monthly reports of health facilities, there are 2,000 pregnant women who attend antenatal care.

### 2.2. Study Population

All pregnant women attending ANC at Debre Markos town public health institutions during the data collection period.

### 2.3. Sample Size and Sampling Procedure

The sample size was determined using a single population proportion formula (precision approach) and with the following assumptions: 50% proportion of PRA since no study done in Ethiopia, 95% level of confidence, and 5% margin of error.

(1) $$\begin{array}{c} n=\frac{{\left(Z\alpha /2\right)}^{2}*p\left(1-p\right)}{d^{2}}=\frac{{\left(1.96\right)}^{2}*0.5\left(1-0.5\right)}{\left(0.05\right)2}=384, \end{array}$$

where *n* is the required sample sizes, *α* is the level of significance, *z* is the standard normal distribution curve value for 95%confidence level = 1.96, *p* is the proportion of PRA, and *d* is the margin of error. Finally, by adding a 10% nonresponse rate, the minimum adequate sample size was 423. All public health facilities in Debre Markos town were considered, and based on the number of ANC case flow among the four public health institutions, proportional allocation of the total sample size was carried out to get the required sample size from each public health facility. Finally, the determined samples were selected by a systematic random sampling technique.

**Figure 1:**
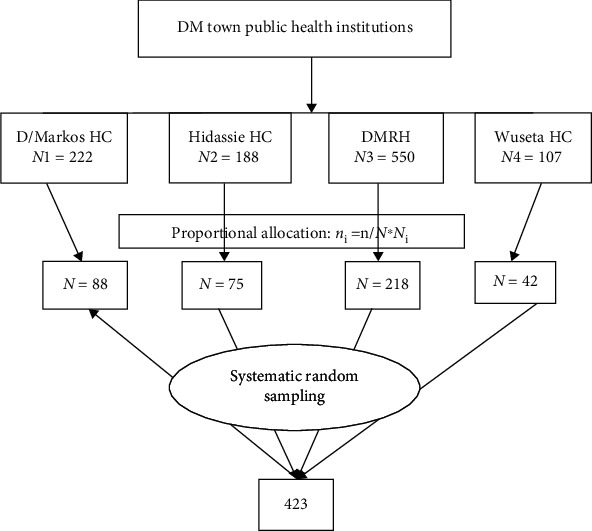
Schematic presentation of the sampling procedure for the study on pregnancy-related anxiety and associated factors among women attending ANC in Debre Markos town public health facilities, northwest Ethiopia, 2021.

The skip interval (*K*) was calculated for each institutions by dividing the estimated average number of women who came for ANC follow-up in each public health institutions during the study period (*Nⱼ*) by the proportionally allocated sample size of each institution (*nⱼ*), and it was the same, *K* = 2.5 (approximated to 3) for all health institutions. The first case was selected randomly using a lottery method. Then, every 3^rd^ unit was taken to get the required sample size from each institution (Figure 1).

### 2.4. Variables

Pregnancy-related anxiety was the dependent variable whereas age of the women, occupational status of the women, educational level of the women, marital status, religion, residence, ethnicity, husband educational level, husband occupation, average household monthly income, family size, gravidity, GA, number of ANC visit, current pregnancy status, age at the 1st pregnancy, previous obstetric complications, history of episiotomy, history of cesarean delivery, depression, social support, history of mental problems, family history of mental problems, intimate partner violence, medical illness, smoking, and alcohol use.

### 2.5. Operational Definitions

#### 2.5.1. Pregnancy-Related Anxiety

Pregnant women who scored ≥13 from the total of score 30 using Pregnancy-Related Anxiety Questionnaire-Revised (PRAQR) were considered pregnancy-related anxiety positive or anxious [12].

#### 2.5.2. Depression

Pregnant women who scored five and above using patient health questionnaire 9 (PHQ-9) were considered depressed [26].

#### 2.5.3. Intimate Partner Violence

Pregnant women screened positive if they answer “yes” to any one of the ranges of sexually, psychologically, and physically or any combination of the three coercive acts used against adult and adolescent women, regardless of the legal status of the relationship with the current intimate partner [27].

#### 2.5.4. Social Support

The Oslow Social Support Scale (OSS-3) scores ranged from 3 to 14 with a score of 3‐8 = poor support, 9‐11 = moderate support, and 12‐14 = strong support [28].

### 2.6. Data Collection Tool and Procedure

The data were collected using a structured, pretested, and interviewer-administered questionnaire through face-to-face interviews. Four BSc and two MSc midwives were recruited for data collection and supervision, respectively. The questionnaire was prepared by reviewing different literatures [5, 6, 8–10, 12, 22, 29–31] and contextualized to the local situations and study objectives. The questionnaire for this study consists of sociodemographic factors, obstetrical and gynecological factors, medical and behavioral factors, psychosocial factors, and the PRAQR.

The outcome variable was measured using PRAQR which has 10 items. Each item has a 4-point Likert scale of 0 = never, 1 = hardly ever, 2 = sometimes, and 3 = yes, quite often, with a cumulative score of 30 points. PRAQR assesses three subscales of anxiety that are specific to pregnancy which are fear of giving birth, fear of bearing a handicapped child, and pregnancy-related concerns about one's physical appearance.

### 2.7. Data Quality Assurance

The questionnaire was first prepared in English and then translated to Amharic (local language) and back to English to maintain its consistency. A pretest was done on 5% of pregnant women who had ANC follow-up outside the study setting (in Finote Selam hospital) to check the wording, order, appropriateness, and feasibility of the tool. Training was given to the data collectors and supervisors for one day on how they collect and record data and about the general aim of the research by the principal investigator. During the actual data collection period, the questionnaire was checked for completeness daily by the supervisors.

### 2.8. Data Processing and Analysis

The collected data were checked manually for completeness and was entered into Epi-data version 4.6 and exported to Statistical Package for Social Sciences (SPSS) version 23. Data coding and recoding were done. Data were checked for errors, outlying observation, missing observation, and inconsistencies. The result of the univariable analysis (descriptive results) was presented as frequencies and percentage. Median and interquartile range (IQR) were used to describe age since it was skewed. Chi-square assumption checked before bivariable analysis. Model fitness also checked by the Hosmer-Lemeshow test. Multicollinearty was checked among variables which had an association with PRA. Variables having a *p* value of ≤0.2 in the bivariable analysis were entered into the multivariable regression analysis. In the multivariable logistic regression model, the AOR with its 95% CI and a *p* value of ≤0.05 were used to declare statistical association. The analyzed data were presented using text, tables, and figures.

## 3. Results

### 3.1. Sociodemographic Characteristics

A total of 408 pregnant women participated in this study giving a response rate of 96.4%. The median age of the respondents was 27 IQR (24, 29) years, and 184 (45.1%) of the respondents were in the age group of 26-30 years. Most of the study participants, 392 (96.1%) were married and 377 (92.4%) were urban dwellers. Nearly half of them, 182 (44.6%) attended college and above in level of education and 184 (45.1%) were house wives in occupation. Regarding husbands' educational level, more than half, 205 (52.3%) were college and above (*n* = 392). One hundred seventy-seven (45.2%) of their husbands were governmental employee (*n* = 392) (Table 1).

### 3.2. Obstetrical and Gynecologic Factors

More than half, 228 (55.9%) of the respondents were multigravida. Among the multigravida women, about sixty-six (28.9%) of the respondents had pregnancy complications in the previous pregnancy. Majority, 347 (85.0%) of the respondents' pregnancy was planned and wanted. About one hundred sixty-three (40.0%) of the respondents were at second trimester of pregnancy (Table 2).

### 3.3. Medical, Behavioral and Psychosocial Factors

About thirty (7.4%) of the respondents had known medical illness which was diagnosed by health care provider/physician. About seven (1.7%) of the respondents had a family with mental illness. Nearly one from eight (13.5%) of the respondents were violated by their intimate partner. More than one-third of participants, 153 (37.5%) had poor social support. Nearly one-third, 115 (28.2%) of participants were depressed (Table 3).

### 3.4. Prevalence of Pregnancy-Related Anxiety among Study Participants

**Figure 2:**
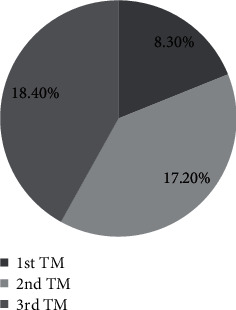
Prevalence of pregnancy related anxiety in trimester among women attending ANC at Debre Markos town public health institutions, Northwest Ethiopia, 2021.

The prevalence of pregnancy-related anxiety among pregnant women attending ANC at Debre Markos town public health institutions was found to be 43.9% (95% CI: 39.5, 49.2) (Figure 2).

### 3.5. Factors Associated with Pregnancy-Related Anxiety

Both bivariable and multivariable logistic regression analyses were done to identify factors associated with pregnancy related anxiety. The factors which had an association with PRA on bivariable analysis were age of the women, residence, educational status of the women, occupation of the women, family size, gravidity, current medical illness reported by physician, intimate partner violence, social support, and depression. However, educational status of the women (no formal education), gravidity (primigravida), encountered intimate partner violence, and social support (poor social support) were significantly associated with PRA in the multivariable analysis.

This study showed that pregnant women who had no formal education were 3.37 (AOR = 3.37; 95% CI: 1.32, 8.58) times more likely to have PRA compared to those women who attended college and above education. This study also revealed that being primigravida increases the odds of PRA by 1.94 (AOR = 1.94; 95% CI: 1.17, 3.24) times compared with multigravida women. Similarly, the odds of having PRA among women who were violated by their intimate partner were 2.88 (AOR = 2.88; 95% CI: 1.47, 5.64) times higher compared to women who did not encounter intimate partner violence. Moreover, pregnant women who had poor social support were two (AOR = 2.05; 95% CI: 1.18, 3.56) times more likely to have PRA compared to those women who had strong social support (Table 4).

## 4. Discussion

This study assessed the prevalence and associated factors of pregnancy related anxiety among women attending ANC at Debre Markos town public health institutions, Northwest Ethiopia, 2021.

The prevalence of pregnancy-related anxiety was found to be 43.9% (95% CI: 39.5, 49.2) as determined using PRAQR. This finding is higher compared to studies done in Tanzania (25%) [12], Soweto, South Africa (15.2%) [11], West Africa (Ghana (11.4%) and Coted'ivoire (17.4%)) [7], Eastern Saudi Arabia (23.6%) [6], Singapore (29.5%) [32], Changchun, China (20.6%) [33], and South Western China (15.04%) [30].

This discrepancy might be due to differences in the study period. This study was done during COVID 19 pandemic. The COVID 19 pandemic increases the risk PRA since it reduces number of ANC visits, social support, and acquisition of information from different sources like neighbors, relatives, families, and other social networks [34, 35].

Another justification for this discrepancy might be difference in study population. This study included pregnant women at all trimesters but other studies included only pregnant women ≤ 32 weeks [12], women at the 1^st^ trimester [11], women at the 3^rd^ trimester [7], women ≥ 38 weeks [33], and women < 15 weeks [30] of gestation. The reason might be that PRA differs with different gestational ages [32]. Pregnant women with advancing gestational age are more likely to develop PRA [10, 19]. In the contrary, PRA is more prevalent at the 1^st^ trimester of pregnancy [36]. Since, this study includes all gestational ages of pregnancy it assessed both extreme effects of trimesters; hence, the magnitude of PRA might be higher.

The difference might be also due to variation in eligibility criteria. For instance, the study done in Singapore included only pregnant women who started the 1st ANC visit at 11 to 14 weeks, whereas this study includes all pregnant women at all ANC visits. Initiation of ANC visit at the early gestational age increases the opportunity of pregnant women to get support, counseling, and appropriate care from the health care providers and which in turn might reduce prevalence of PRA.

This result is in line with studies conducted in Parakou, Benin (44.9%) [10], South of Minas Gerais, Brazil (42.9%) [8], Lahore, Pakistan (49%) [31], and Dhulikhel Hospital, Nepal (46.4%) [37].

On the other hand, this finding is lower compared with those of studies conducted in Bangalore, Southern India (55.7%) [13], China (59.07%) [38], and East Delhi, India (63%) [1]. The discrepancy with a study done in Bangalore, Southern India [13], might be due to difference in participants' characteristics like age and pregnancy status in which 30% of the study participants were ≤20 years, whereas only 7.6% of this study participants were ≤20 years old and 42.2% of their participants' pregnancy was unplanned while 85.0% of participants' pregnancy in this study was planned. Younger age increases the risk of PRA [37], and women having unplanned pregnancy are more likely to develop anxiety [6, 9, 31]. Hence, PRA might be lower in this study.

The discrepancy with a study done in China [38] might be due to difference in study population which was done on pregnant women with gestational diabetes mellitus who are more vulnerable groups to PRA due to the additional negative effect of this medical condition on maternal psychological perception and might result in higher PRA.

The difference with a study done in East Delhi, India [1], might be due to difference in study setting that was a community-based study. The prevalence of PRA might be lower in an institutional-based study compared to a community-based study, since women with better health seeking behavior coming to health facilities while community-based study comprises all pregnant women.

In this study, maternal educational status was significantly associated with PRA. Pregnant women who had no formal education had more than three times higher odds of PRA than study participants who attended college and above. This finding is supported by studies done in China [33] and Pakistan [31]. The possible justification might be a lower level of education associated with lower socioeconomic status, financial dependency, unemployment, and more daily troubles [39]. Those in turn decrease women's participation in social activities, information attainment, and getting appropriate care. Hence, mental health deteriorates and might result in higher prevalence of PRA.

Gravidity of the women was another factor associated with PRA. Pregnant women who were primigravida experienced PRA 1.94 times higher compared with their counter parts. This finding is supported by a study done in Nepal [40] and South western China [30]. The possible justification might be primigravida women are more prone to fear of giving birth because they did not have previous labor experience [41]. Thus, this fear of child birth increases the psychological vulnerability of the women to PRA due to higher expectations of labor pain, lack of experience in becoming a mother, sense of new life and adding of demand and responsibility [42].

Similarly, intimate partner violence was associated with PRA. The odds of having PRA among pregnant women who were violated by their intimate partner were 2.88 times higher than their counter parts. This finding is supported by a study done in Nigeria [9]. The possible justification might be intimate partner violence is unfavorable external factor which affects the physical, social and psychological health of the women. Hence, sense of being injured and valueless give rise to poor mental health [43].

In this study, social support was also the significantly associated variable. Pregnant women who had poor social support experienced PRA two times higher than pregnant women who had strong social support. This finding is supported by studies done in India [13], China [30], and Pakistan [31]. This might be due to the fact that social support is a process of interaction between subjective and objective support of people in various aspects such as information, tool, and emotional support from many sources including family, friends, neighbors, colleagues, and groups and poor social support can lead to a sense of isolation and loneliness and those in turn could rise to PRA [44]. Improved supports of pregnant women from family, neighbor, or health care providers might be encouraging to strengthen women's belief as childbirth is a physiological and controllable process and reduce the PRA.

## 5. Limitation of the Study

Social desirability bias might be there in some variable measurement like intimate partner violence. A private room was used during interviewing of the study participants to minimize social desirability bias. Recall bias might be introduced and probing was used for increasing the participants' ability to remember.

## 6. Conclusion

This study showed that pregnancy-related anxiety was prevalent among pregnant women attending ANC at Debre Markos town public health institutions. Pregnant women having no formal education, primigravida, encountered intimate partner violence, and poor social support had increased risk of pregnancy-related anxiety.

To tackle this problem, giving attention to those women with the identified risk factors is important. Empowering women through education, awareness creation, partner counseling, and enhanced social support are better to prevent pregnancy-related anxiety. It is better to identify PRA early by health-care professionals to provide cognitive behavioral therapy and support for their psychological health. The authors also suggest for researchers to conduct further qualitative studies that cover a wider setting at different areas about PRA.

## Figures and Tables

**Table 1:** Sociodemographic characteristics of women attending antenatal care at Debre Markos town public health institutions, Northwest Ethiopia, 2021 (*n* = 408).

Variable	Frequency	Percentage
*Age of the women*		
≤20	31	7.6
21-25	133	32.6
26-30	184	45.1
>30	60	14.7
*Marital status of the women*		
Married	392	96.1%
Unmarried	16	3.9%
*Husband educational level (n* = 392)		
No formal education	35	8.9%
Primary (1–8)	51	13.0%
Secondary (9–12)	101	25.8%
College and above	205	52.3%
*Husband occupation (n* = 392)		
Farmer	11	2.8%
Private employee	137	34.9%
Government employee	177	45.2%
Merchant	67	17.1%
*Religion of the woman*		
Orthodox	390	95.6%
Muslim	11	2.7%
Protestant	7	1.7%
*Residence*		
Urban	377	92.4%
Rural	31	7.6%
*Ethnicity*		
Amhara	406	99.5%
Oromo	2	0.5%
*Educational level of the woman*		
No formal education	34	8.3%
Primary (1–8)	68	16.7%
Secondary (9–12)	124	30.4%
Collage and above	182	44.6%
*Occupation of the woman*		
House wife	184	45.1%
Private employee	49	12.0%
Government employee	108	26.5%
Merchant	67	16.4%
*House hold average monthly income*		
≤3000	165	40.4%
>3000	243	59.6%
*Family size*		
≤3	327	80.1%
≥4	81	19.9%

**Table 2:** Obstetrical and gynecologic characteristics of women attending antenatal care at Debre Markos town public health institutions, Northwest Ethiopia, 2021 (*n* = 408).

Variable	Frequency	Percentage
*Gravidity*		
Primigravida	180	44.1%
Multigravida	228	55.9%
*Age at first pregnancy*		
<18	30	7.4%
≥18	378	92.6%
*History of obstetric complications (n* = 228)		
Yes	66	28.9%
No	162	71.1%
*Type of obstetric complications (n* = 66)		
Abortion	32	48.4%
Pregnancy induced hypertension	10	15.2%
Still birth	24	36.4%
*History of episiotomy (n* = 228)		
Yes	72	31.6%
No	156	68.4%
*History of cesarean birth (n* = 228)		
Yes	27	11.8%
No	201	88.2%
*Status of current pregnancy*		
Planned and wanted	347	85.0%
Unplanned and unwanted	6	1.5%
Unplanned but wanted	55	13.5%
*Time of pregnancy (GA)*		
First trimester	85	20.8%
Second trimester	163	40.0%
Third trimester	160	39.2%
*Number of ANC visit*		
1-3	323	79.2%
≥4	85	20.8%

**Table 3:** Medical, behavioral, and psychosocial characteristics of women attending antenatal care at Debre Markos town public health institutions, Northwest Ethiopia, 2021 (*n* = 408).

Variable	Frequency	Percent
*Current medical illness*		
Yes	30	7.4%
No	378	92.6%
*Type of medical illness (n* = 30)		
Hypertension	6	20.0%
DM	9	30.0%
HIV	15	50.0%
*Smoking*		
No	408	100%
*Alcohol use currently*		
Yes	103	25.2%
No	305	74.8%
*Frequency of alcohol use (n* = 103)		
Daily	2	1.9%
1-2 times/week	51	49.5%
1-3 times/month	39	37.9%
<1 times in a month	11	10.7%
*History of mental illness*		
No	408	100%
*Family with mental illness*		
Yes	7	1.7%
No	401	98.3%
*Intimate partner violence*		
Yes	55	13.5%
No	353	86.5%
*Social support*		
Poor support	153	37.5%
Moderate support	144	35.3%
Strong support	111	27.2%
*Depression*		
Yes	115	28.2%
No	293	71.8%

**Table 4:** Bivariable and multivariable logistic regression analysis of factors associated with pregnancy related anxiety among women attending antenatal care at Debre Markos town public health institutions, Northwest Ethiopia, 2021 (*n* = 408).

Variable	Pregnancy related anxiety		COR (95% CI)	AOR (95% CI)
	Yes	No		
*Age of women*				
≤20	16	15	1	1
21-25	67	66	0.94 (0.43, 2.08)	1.12 (0.47, 2.63)
26-30	63	121	0.48 (0.22, 1.05)	0.85 (0.35, 2.06)
>30	33	27	1.14 (0.48, 2.73)	2.05 (0.68, 6.14)
*Residence*				
Urban	160	217	1	1
Rural	19	12	2.14 (1.01, 4.55)	1.64 (0.71, 3.79)
*Educational level of the women*				
No formal education	24	10	4.11 (1.85, 9.13)	3.37 (1.32, 8.58)^∗^
Primary (1–8)	36	32	1.93 (1.01, 3.39)	1.90 (0.98, 3.66)
Secondary (9–12)	52	72	1.24 (0.77, 1.97)	1.20 (0.68, 2.10)
College and above	67	115	1	1
*Occupation of the women*				
Housewife	88	96	0.88 (0.50, 1.55)	0.92 (0.49, 1.72)
Private employee	17	32	0.51 (0.24, 1.10)	0.45 (0.19, 1.05)
Government employee	40	68	0.57 (0.30, 1.05)	0.76 (0.35, 1.66)
Merchant	34	33	1	1
*Family size*				
≤3	149	178	1	1
≥4	30	51	0.70 (0.42, 1.15)	0.76 (0.41, 1.41)
*Gravidity*				
Primigravida	95	85	1.91 (1.28, 2.85)	1.94 (1.17, 3.24)^∗∗^
Multigravida	84	144	1	1
*Current medical illness reported by physician*				
Yes	17	13	1.74 (0.82, 3.69)	1.47 (0.59, 3.64)
No	162	216	1	1
*Intimate partner violence*				
Yes	37	18	3.05 (1.67, 5.57)	2.88 (1.47, 5.64)^∗∗^
No	142	211	1	1
*Social support*				
Poor support	86	67	2.37 (1.43, 3.92	2.05 (1.18, 3.56)^∗∗^
Moderate support	54	90	1.10 (0.66, 1.85)	1.05 (0.59, 1.85)
Strong support	39	72	1	1
*Depression*				
Yes	58	57	1.44 (0.93, 2.23)	1.34 (0.81, 2.19)
No	121	172	1	1

AOR: adjusted odds ratio; COR: crude odds ratio; CI: confidence interval; 1: reference category; ^∗^*p* ≤ 0.05; ^∗∗^*p* ≤ 0.01.

## Data Availability

The data used for this research analysis are available in the corresponding author upon request.

## Abbreviations

ANC: Antenatal care
AOR: Adjusted odds ratio
BSc: Bachelor of Science
CI: Confidence interval
COR: Crude odds ratio
GA: Gestational age
MSc: Master of science
PRA: Pregnancy-related anxiety
PRAQR: Pregnancy Related Anxiety Questionnaire-Revised
STAI: State Trait Anxiety Inventory
SPSS: Statistical Package for Social Science.
